# Effects of an education program on knowledge and self-perception of school personnel in preparing to care for type 1 diabetes students

**DOI:** 10.31744/einstein_journal/2020AO5101

**Published:** 2020-02-19

**Authors:** Maria dos Anjos Coelho Rodrigues Dixe, Clementina Maria Gomes de Oliveira Gordo, Helena Borges Pereira Catarino, Teresa Kraus, Eva Patricia da Silva Guilherme Menino

**Affiliations:** 1 Center for Innovative Care and Health Technology Escola Superior de Saúde Instituto Politécnico de Leiria LeiriaPT Portugal Center for Innovative Care and Health Technology , Escola Superior de Saúde , Instituto Politécnico de Leiria , Leiria , PT , Portugal .; 2 Escola Superior de Enfermagem de Coimbra CoimbraPT Portugal Escola Superior de Enfermagem de Coimbra , Coimbra , PT , Portugal .

**Keywords:** Diabetes mellitus, Education, Knowledge, Child, Adolescent

## Abstract

**Objective:**

To assess the academic and professional background of school personnel; to assess the impact of the Diabetes + Support given by School Personnel to Children with Type 1 Diabetes Program on the school personnel’s knowledge and confidence to support students with type 1 diabetes; to compare their level of knowledge with the academic and professional variables of the school personnel.

**Methods:**

A quasi-experimental pre-test/post-test study design without a Control Group. Study with a sample of 129 (before intervention – T0) and 113 (after intervention – T1) pre-school to secondary school personnel from participating schools, with at least one student with type 1 diabetes. The project was approved by the Ethics Committee of the Portuguese Ministry of Education.

**Results:**

Most school personnel included in the study were teachers (51.2%). After training, they were more confident than before to support children with type 1 diabetes (p<0.05). Regarding knowledge levels, the differences between T0 (10.8±2.8; P _50_ =11) and T1 (13.7±2.1; P _50_ =11) were statistically significant (p<0.001). Of the 113 school personnel who participated in the final assessment, 89 (78.85%) increased their level of knowledge.

**Conclusion:**

The program was effective to enhance knowledge and boost confidence to support students with diabetes.

## INTRODUCTION

Type 1 *diabetes mellitus* due to autoimmune b-cell destruction, usually leading to absolute insulin deficiency, ^1^ is one of the most common childhood endocrine and metabolic diseases. ^2^ The number of cases has been increasing globally and, currently, more than half a million children under the age of 14 are affected, ^3^ therefore there is a need to monitor these children and young people in all aspects of their lives and, particularly, at school.

Several authors ^4 , 5^ have already identified problems in the support given to type 1 diabetes management in a school setting, often related to the poor training of school personnel to deal with diabetes emergencies, such as hypo- or hyperglycemia episodes. These authors also identified the absence of individual health plans, an insufficient number of school nurses or school staff trained to meet diabetes-related needs, and the unsuitability of nutritional services or nutritional information provided to parents by the school, with a view to planning the required insulin doses.

These children actually need school personnel to have basic knowledge and competencies to provide a safe school setting. ^6^ Parents and the healthcare team responsible for these children should work together to provide the school system and other carers with the information required, so that they can participate fully and safely in all educational/leisure settings. ^7^

To ensure their safety and to provide support to these diabetic children/young people, schools must be equipped with a series of policies, namely: if there are one or more diabetic students, schools must ensure that at least two members of staff are trained to provide support; ensure there is an area for diabetes self-care, including blood glucose monitoring and respect for their privacy; develop of an individual health plan for each student that is discussed with the parents, the school headmaster and personnel; and each teacher or supervisor must know how to recognise and treat hypoglycaemia. ^1 , 8 , 9^

## OBJECTIVE

To assess the impact of the Diabetes + Support given by School Personnel to Type 1 Diabetes Children (DARE+) Program on the school personnel’s knowledge and confidence levels to support students with type 1 diabetes; to compare their level of knowledge with the academic and professional variables of the school personnel.

## METHODS

This study used a pre-test post-test quasi-experimental design, with no Control Group.

The sample comprised school personnel who worked with type 1 diabetic children. Of the total of 12 participating schools, 129 school staff members participated at the beginning of the study (T0) and 113 at the end of the study (T1). The data were collected from March 2014 to March 2015.

The intervention involved the following stages:

- Since the training of the school personnel was conducted by school nurses, the first stage of the project included capacity-building of the latter. This training lasted 21 hours, over 3 days, with 14 hours focusing on children/adolescents with type 1 diabetes. ^9 , 10^
- Preliminary meeting with the person in charge of the school cluster to present the objectives and obtain authorization schedule the intervention.
- DARE+ school intervention programme, encompassing two sessions lasting a total of 6 hours, 1 week apart. Each session included a theory-based and a practical-based section, and the topics recommended by national and international associations were addressed. ^1 , 9 , 10^

As part of this intervention and to implement the individual health plan, which comprises the elements indicated by the American Diabetes Association (ADA) ^10^ and *Direção Geral da Saúde* (DGS) (Health General Office of the Ministry of Health), ^9^ a meeting was run with the parents of children/young people and the school personnel in charge of providing them with support.

The data were collected via a three-part self-completed questionnaire:

- Academic and professional background of the school personnel: profession, academic qualifications, years of professional experience, years of professional experience at the institution and type of employment contract.
- Diabetes knowledge questionnaire. ^11^ This instrument comprises 17 items with three possible answers (only one of which is correct) and a fourth option “Don’t know”.

After receiving authorisation from the authors the questionnaire was linguistically and culturally validated in accordance with international guidelines. ^12 - 14^ Internal consistency was also determined by calculating Cronbach’s alpha, which was recorded as 0.926. A score of 1 was attributed to the correct answer and zero to incorrect answers and “Don’t know” responses. The score can thus fluctuate between zero and 17 points.

- Confidence of school personnel in supporting students with type 1 diabetes questionnaire. ^11^ This scale comprises a 4-point Likert scale with five possible answers (1 for I completely disagree; 2 for I disagree; 3 for no opinion; 4 for I agree, and 5 for I completely agree). The values can fluctuate from 4 to 20, and the higher the value, the greater the level of confidence to support students with type 1 diabetes.

The psychometric characteristics were determined and a Cronbach’s alpha value of 0.836 calculated.

Construct validity was tested using exploratory factor analysis with a Varimax rotation with Kaiser normalization. Only one factor was obtained, which explains the 67.4% variance. Communality values fluctuate between 0.670 and 0.714. The Kaiser-Meyer-Olkin is 0.799, and Bartlett’s sphericity test value is 200.677 (p<0.001).

Statistical analysis was performed using the (SPSS), version 21.0 software. Relative and absolute weaknesses were used for the academic and professional background of the school personnel.

Spearman correlation (independent test) and Wilcoxon (paired test) nonparametric statistics were used because the data did not have a normal distribution (assessed by the Kolmogorov-Smirnov test). As not all the groups have an n≥30, the use of the central limit theorem is not permitted.

A level of significance of 0.05 (p<0.05) was adopted in inferential analyses.

This research project was approved by the Research Ethics Committee of the Ministry of Education (Case no. 0396300001) and an Informed Consent was obtained from all individual participants enrolled in the study.

## RESULTS

Given that each in programme and at each school the intervention involved small groups of personnel, the choice was made not to ask about the age and the sex of the participants. The reason for this was two-fold: not being essential for the study objectives and preventing the personnel from being identified, because in some schools only one male member of staff participated.

More than half (51.2%) of the participants were teachers, of whom 13.6% held a Master’s/graduate degree and had been working for more than 10 years at the organization, and had a contract with them ( Table 1 ).

**Table 1:** Academic and professional background in the first period (T0)

Academic and professional variables	n (%)
Profession	
Operational assistant	42 (32.6)
Teacher	66 (51.2)
Other (monitor, driver, infant teacher, cook)	21 (16.3)
Academic qualifications, years	
6	15 (12.0)
9	11 (8.8)
12	20 (16.0)
Bachelor’s degree/Degree	62 (49.6)
Master’s/Graduate degree	17 (13.6)
Professional experience, years	
<1	7 (5.6)
1-5	17 (13.6)
6-9	22 (17.6)
10-20	39 (31.2)
>20	40 (32.0)
Years at institution	
<1	13 (10.2)
1-5	30 (23.6)
6-9	25 (19.7)
10-20	34 (26.8)
>20	25 (19.7)
Employment details	
Fixed-term full time contract	108 (83.8)
Permanent contract	2 (1.6)
Part-time contract	10 (7.8)
Other	9 (7.0)

Considering that the higher the value, the greater the school personnel’s confidence level to support type 1 diabetic students, on average after training (x̄;=16.2 ±1.3; P _50_ =16), the school personnel perceived their confidence level to be better than before (x̄=9.8±3; P _50_ =9.8) to support type 1 diabetes children. This difference was significant (Wilcoxon=-9.536; p<0.001). Only ten participants did not improve their confidence level, eight remained the same and two registered lower values.

Regarding the level of knowledge, it was found that the differences between T0 (x̄=10.8±2.8; P _50_ =11) and T1 (x̄=13.7±2.1; P _50_ =11) were significant (Wilcoxon=-7.914; <0.001) and 89 of the school personnel increased their level of knowledge. Of the 113 school personnel that participated in the final assessment, 89 (78.85%) increased their level of knowledge.

An analysis of the relation between the level of knowledge and level of confidence among school personnel to support type 1 diabetes students before training showed there was a low, positive and significant correlation between the two variables (rs=220; p<0.05). As the level of knowledge increased, so did the level of confidence to support diabetic children/young people. After training, the correlation was similarly weak, negative but not significant (rs=-0.016; p>0.05).

Both before (χ ^2^ =11.6; p<0.05) and after (χ ^2^ =13.1; p<0.05) the intervention, the level of knowledge differed according to academic qualifications. The higher the academic qualifications, the greater was the personnel’s level of knowledge regarding type 1 diabetes ( Table 2 ).

**Table 2:** Application of the Wilcoxon test to the level of knowledge of type 1 diabetes among personnel, before and after the intervention, according to academic qualifications

Academic qualifications	Before the intervention			After the intervention			Z	p value
	x̄	Standard deviation	Median	x̄	Standard deviation	Median		
Year 6	9.7	2.5	9	11.9	3.3	13	-2.177	0.029
Year 9	8.0	4.2	10	13.0	1.8	13	-2.613	0.009
Year 12	10.9	2.1	10.5	13.6	1.8	14	-2.899	0.004
Bachelor’s degree/Further education	11.4	2.3	12	14.2	1.7	15	-5.831	0.000
Graduate/master’s degree	11.5	3.0	12	14.6	1.5	15	-2.662	0.008

A division of the sample according to years of professional experience showed that before the intervention the level of knowledge was not related to the number of years of professional experience (p>0.05). After the intervention, the level of knowledge (p<0.05) differed according to the number of years of professional experience.

The greater the number of years of experience, the better the level of knowledge was, apart from groups of professionals with less that 1 year of experience. Among this group, an increase in the level of knowledge was not observed ( Table 3 ).

**Table 3:** Application of the Wilcoxon test to the level of knowledge of type 1 diabetes among personnel before and after the intervention, according to years of professional experience

Years of professional experience	Before the intervention			After the intervention			χ ^2^	p value
	x̄	Standard deviation	Median	x̄	Standard deviation	Median		
<1	10.7	2	11	11.2	3.5	11	-0.816	0.414
1-5	11	2.7	12	12.8	1.9	13	-2.408	0.016
6-9	10.8	2.1	11	14	1.6	14	-3.899	0.000
10-20	10.7	2.6	11	13.9	1.9	14	-4.494	0.000
>20	11.3	2.9	11.5	14.4	1.6	14	-4.582	0.000

## DISCUSSION

When a child with type 1 diabetes is included in a school, it is important for the institution to orientate and manage its action plan around two key points: the training of personnel and the development and application of the child’s individual health plan. ^8 - 10^ This study also focused on these two key points.

The integration of primary care and hospital professionals, and cooperation among them, was also one of the concerns when defining the intervention to be implemented, as suggested several authors. ^9 , 15^ Prior to the intervention, the school nurses underwent training involving the multiprofessional pediatric team from a hospital and the multiprofessional primary care team.

The school nurses participated in the intervention phase alongside the school personnel. This intervention lasted 6 hours, a longer number of hours than the 4 hours recommended by the DGS. ^9^

All school personnel took part in the intervention, namely teachers, infant teachers, operational assistants, drivers, cooks and cultural entertainers, who stated that diabetes education must target not only teachers but all staff who interact with children. ^9 , 10^

More than half the sample (63.2%) held higher education degrees. The same percentage of personnel had more than 10 years of experience.

In a literature review, ^2^ most children were found not to be receiving the necessary care while at school. Wagner et al., ^16^ had already come to this conclusion, and when analysing 132 school personnel found that they were not prepared to care for diabetic children/young people.

Although the studies by Alnasir ^17^ and Aycan et al., ^18^ were only conducted with teachers, the findings confirmed the low level of knowledge of the samples to support diabetic children. By separating the level of knowledge into areas, others authors ^6^ found that knowledge of symptoms was good, however knowledge of complications and diabetes management was poor. Some of these points were included in the DARE+ intervention programme.

If in order to provide proper care, knowledge is required, and the level of knowledge of the participants in this study, before the intervention, was 63.7% of maximum score possible. These values are slightly higher than those presented in other studies, ^17 , 18^ even though these investigations were conducted only with a sample of teachers. After the DARE+ programme intervention, it was found that the level of knowledge of the school personnel improved, achieving 80.6% of maximum possible score.

The guidelines from the *Direção Geral da Saúde* state that very young children need more care, and schools whose personnel are unprepared may be at a disadvantage. Therefore, school nurses need to work in partnership with children, parents and school personnel to guarantee that the treatment/monitoring of diabetic children/young people is fully integrated into the daily life of the school. ^9^ The studies show that primary school personnel are concerned about the administration of insulin injections and monitoring of blood glucose values, ^19^ hence training school personnel optimises the safety of diabetic children. ^19 - 22^

After the intervention, the school personnel were more confident than before in supporting children with type 1 diabetes and the difference was statistically significant (p<0.01). Other studies ^20 - 22^ also showed enhanced knowledge of school personnel about caring of type 1 diabetic students achieved through an education programme.

In contrast, Husband et al., ^11^ did not find enhanced knowledge of teachers, when using a non-interactive CD-ROM training methodology. They recommended the use of more active methodologies in the intervention. These data reinforced the decision in this study to use active methodologies, with the participation of the school personnel in drills and case discussions.

Before the intervention, the level of knowledge was not related to the number of years of professional experience (p>0.05). Wagner et al., ^16^ also found that school personnel with 10 years of professional experience, and in contact with diabetic children/young people were not prepared to care for them. Participation in the DARE+ intervention programme led to enhanced level of knowledge of school personnel (p<0.05). Only the group of professionals with less than 1 year of experience did not experience a gain in the level of knowledge.

Both before (χ ^2^ =11.6; p<0.05) and after the intervention, the level of knowledge (χ ^2^ =13.1; p<0.05) differs according to academic qualifications. The higher the academic qualifications, the greater the level of knowledge of the personnel about type 1 diabetes.

It is important to emphasize that in all schools an individual health plan was defined/improved for each child/young person, allowing them all to have a key member of staff to support them with diabetes care during school hours. Although this is considered good practice and is recommended, ^1 , 8 - 10 , 23^ it does not represent the reality, since data from a study conducted by Driscoll et al., ^22^ in the United States, showed that 57% of children did not have any school personnel in charge of monitoring their diabetes care during school hours.

Apart from assessing the knowledge and confidence of school personnel in the short, medium and long term, it is necessary to assess the effect on children, especially with regards to quality of life, number of incidents, quality of academic life, academic performance and school absenteeism.

It is also necessary to be mindful of the continued education/training of the nurses that instruct the school personnel, and also the school personnel themselves, since they provide direct and immediate care to children in emergencies.

In this field and because the implementation of plans involving the key stakeholders is essential, it is not easy to conduct randomised studies; however, they are important to validate not only the intervention programme, but the resources involved in the programme.

The study findings reinforce the need to develop recommendations and policies to healthcare services, and particularly school health services to implement the training of school personnel, since it has been established that structured actions focusing on the training of personnel increased their knowledge and perception of improvement in their response to type 1 diabetic students.

The definition of a structured program with evaluation, and now with these results, which reinforce the efficacy of the program, may influence/improve practices as a standard plan of integrated action is defined, involving all the key elements: family, students, team of carers from the speciality field, school health team and school.

This study contributes to the field of research for identifying integrated support strategies for children and young people with type 1 diabetes. New findings raise new issues relating to in-school care for students with type 1 diabetes, and corroborate previous research, fostering new studies to clarify which strategies work best in practice among different groups, and in different circumstances.

The limitations of this study comprise sample size, recruitment mode and limited geographical location of the sample. Experimental studies with a Control Group and with larger and randomized samples are warranted.

Another limitation is there was no follow-up of the school personnel, or analysis of the impact on gains in the quality of life of student and family, especially regarding the major indicators revealing better disease management and consequently better care for diabetic children/young people.

## CONCLUSION

The Diabetes + Support given by School Personnel to Type 1 Diabetes Children (DARE +) intervention was effective to enhance knowledge and self-perception of the preparation of school personnel to support diabetic children/young people and it is important to highlight some suggestions and implications for practice and research.

Carrying out interventions to enhance knowledge and confidence is important in the short-term. Appropriate follow-up is, therefore, recommended, in addition to training and continued support for school personnel from nurses, thus allowing for constant recycling of knowledge on the issue.

## References

[1] 1. American Diabetes Association. Children and adolescents. Diabetes Care. 2017;40(Suppl 1):S105-13. 10.2337/dc17-S015 27979899

[2] 2. Marks A, Wilson V, Crisp J. The management of type 1 diabetes in primary school: review of the literature. Issues Compr Pediatr Nurs. 2013;36(1-2): 98-119. Review. 10.3109/01460862.2013.782079 23597278

[3] 3. International Diabetes Federation (IDF). Kids and diabetes in school (KIDS) [Internet]. Brussels, Belgium; IDF [cited 2018 Jan 11]. Available from: https://www.idf.org/our-activities/education/kids-project.html

[4] 4. Amillategui B, Calle JR, Alvarez MA, Cardiel MA, Barrio R. Identifying the special needs of children with Type 1diabetes in the school setting. An overview of parents’ perceptions. Diabet Med. 2007;24(10):1073-9. 10.1111/j.1464-5491.2007.02250.x 17888130

[5] 5. Schwartz FL, Denham S, Heh V, Wapner A, Shubrook J. Experiences of children and adolescents with type 1 diabetes in school: survey of children, parents and schools. Diabetes Spectr. 2010;23(1):47-55.

[6] 6. Al Duraywish AA, Abdelsalam MN. Assessment of the primary and intermediate school staffs’ knowledge, attitude and practice on care of children with type 1 diabetes at school. Sudan Journal of Medical Sciences (SJMS). 2017;12(1):33-45.

[7] 7. American Diabetes Association. Diabetes care in the school and day care setting. Diabetes Care. 2014;37(Suppl 1): S91-6. 10.2337/dc14-S091 24357216

[8] 8. Lawrence SE, Cummings EA, Pacaud D, Lynk A, Metzger DL. Managing type 1 diabetes in school: recommendations for policy and practice. Paediatr Child Health. 2015;20(1):35-44. English, French. 10.1093/pch/20.1.35 PMC4333753 25722642

[9] 9. Direção Geral da Saúde (DGS). Crianças e Jovens com Diabetes Mellitus Tipo 1 na Escola [Internet]. Lisboa: DGS; 2016 [cited 2018 Jan 18]. Disponível em: https://www.dge.mec.pt/sites/default/files/Esaude/orientacao_diabetes_dez2016_assinada.pdf

[10] 10. Jackson CC, Albanese-O’Neill A, Butler KL, Chiang JL, Deeb LC, Hathaway K, et al. Diabetes care in the school setting: a position statement of the American Diabetes Association. Diabetes Care. 2015;38(10):1958-63. Review. 10.2337/dc15-1418 26404925

[11] 11. Husband A, Pacaud D, Grebenc K, McKiel E. The effectiveness of a CD-ROM in educating teachers who have a student with diabetes. Diabetes Research and Clinical Practice. 2000;50(Suppl 1):286-90.

[12] 12. Guillemin F. Cross-cultural adaptation and validation of health status measures. Scand J Rheumatol. 1995;24(2):61-3. 10.3109/03009749509099285 7747144

[13] 13. Beaton DE, Bombardier C, Guillemin F, Ferraz MB. Guidelines for the process of cross-cultural adaptation of self-report measures. Spine (Phila Pa 1976). 2000;25(24):3186-91. Review. 10.1097/00007632-200012150-00014 11124735

[14] 14. Rahman A, Igbal Z, Waheed W, Hussain N. Translation and cultural adaptation of health questionnaires. J Pak Med Assoc. 2003;53(4):142-7. 12776898

[15] 15. Pansier B, Schulz PJ. School-based diabetes interventions and their outcomes: a systematic literature review. J Public Health Res. 2015;4(1):467. Review. 10.4081/jphr.2015.467 PMC4407044 25918699

[16] 16. Wagner J, James A. A pilot study of school counselor’s preparedness to serve students with diabetes: relationship to self-reported diabetes training. J Sch Health. 2006;76(7):387-92. 10.1111/j.1746-1561.2006.00130.x 16918873

[17] 17. Alnasir FA. Assessment of Knowledge of Diabetes Mellitus among Bahraini School Teachers. Bahrain Med Bull. 2003;25(4):1-8.

[18] 18. Aycan Z, Önder A, Çetinkaya S, Bilgili H, Yıldırım N, Baş VN, et al. Assessment of the knowledge of diabetes mellitus among school teachers within the scope of the managing diabetes at school program. J Clin Res Pediatr En docrinol. 2012;4(4):199-203. 10.4274/Jcrpe.756 PMC3537286 23032146

[19] 19. Boden S, Lloyd CE, Gosden C, Macdougall C, Brown N, Matyka K. The concerns of school staff in caring for children with diabetes in primary school. Pediatric Diabetes. 2012;13(6):e6-13. 10.1111/j.1399-5448.2011.00780.x 21595805

[20] 20. Siminerio LM, Koerbel G. A diabetes education program for school personnel. Pract Diab Int. 2000;17(6):174-7.

[21] 21. Smith CT, Chen AM, Plake KS, Nash CL. Evaluation of the impact of a diabetes education curriculum for school personnel on disease knowledge and confidence in caring for students. J Sch Health. 2012;82(10):449-56. 10.1111/j.1746-1561.2012.00721.x 22954163

[22] 22. Driscoll KA, Volkening LK, Haro H, Ocean G, Wang Y, Jackson CC, et al. Are children with type 1 diabetes safe at school? Examining parent perceptions. Pediatr Diabetes. 2015;16(8):613-20. 10.1111/pedi.12204 25266418

[23] 23. Kaufman F, Jackson C, Bobo N; National Nursing Education Program. Health care plans to manage diabetes at school. NASN Sch Nurse. 2010;25(6):276-8. 10.1177/1942602X10381786 21121424

