# Supplementary material for: Effects of an education program on knowledge and self-perception of school personnel in preparing to care for type 1 diabetes students
Source: Einstein (Sao Paulo). 2020 Feb 19;18:eAO5101. doi: 10.31744/einstein_journal/2020AO5101 (PMC7032886; doi:10.31744/einstein_journal/2020AO5101)
Supplement: Translation

# Translation: Efeitos de um programa de educação nos conhecimentos e na autopercepção dos educadores escolares na preparação para cuidar de crianças diabéticas tipo 1

**Authors:** Maria dos Anjos Coelho Rodrigues Dixe, Clementina Maria Gomes de Oliveira Gordo, Helena Borges Pereira Catarino, Teresa Kraus, Eva Patricia da Silva Guilherme Menino

**Affiliations:** 1 Portugal Center for Innovative Care and Health Technology, Escola Superior de Saúde, Instituto Politécnico de Leiria, Leiria, PT, Portugal .; 2 Portugal Escola Superior de Enfermagem de Coimbra, Coimbra, PT, Portugal .

**Keywords:** Diabetes mellitus, Educação, Conhecimento, Criança, Adolescente

## Abstract

**Objetivo:**

Avaliar a formação acadêmica e profissional dos educadores escolares; avaliar o impacto do Diabetes + Apoio dado pelo Programa Responsáveis Escolares pelas Crianças com Diabetes Tipo 1 no nível de conhecimento e de confiança dos educadores escolares, para apoiar os alunos com diabetes tipo 1; comparar o nível de conhecimento dos educadores escolares com suas variáveis acadêmicas e profissionais.

**Métodos:**

Estudo quasi-experimental, do tipo pré-teste/pós-teste sem Grupo Controle, realizado em amostra de 129 (antes da intervenção – T0) e 113 (pós-intervenção – T1) educadores escolares, da pré-escola ao ensino secundário, que trabalhavam em escolas com pelo menos uma criança/jovem com diabetes tipo 1. O projeto foi aprovado pelo Comitê de Ética do Ministério da Educação de Portugal.

**Resultados:**

A maioria dos educadores escolares incluídos no estudo era professor (51,2%). Após a intervenção, os educadores escolares sentiram-se mais confiantes do que antes, para apoiar crianças com diabetes tipo 1 (p<0,05). Em relação aos níveis de conhecimento, as diferenças entre T0 (10,8±2,8; P_50_=11) e T1 (13,7±2,1; P_50_=11) foram estatisticamente significantes (p<0,001). Dos 113 educadores escolares que participaram da avaliação final, 89 (78,85%) aumentaram seu nível de conhecimento.

**Conclusão:**

O programa foi eficaz para aumentar o conhecimento e a confiança dos educadores escolares para apoiar as crianças/jovens com diabetes tipo 1.

## INTRODUÇÃO

O *diabetes mellitus* tipo 1, decorrente da destruição autoimune de células beta levando à deficiência absoluta de insulina,^1^ é uma das doenças endócrinas e metabólicas mais comuns da infância.^2^ O número de casos vem aumentando em todo o mundo, e atualmente, mais de meio milhão de crianças com menos de 14 anos têm a doença,^3^ sendo necessário acompanhar essas crianças e jovens em todos os aspectos de sua vida, principalmente na escola.

Diversos autores^4 , 5^ já identificaram problemas no apoio oferecido para controle do diabetes tipo 1 no ambiente escolar, geralmente relativos ao treinamento inadequado dos educadores escolares para lidar com emergências decorrentes do diabetes, como episódios de hipo- ou hiperglicemia. Esses autores também identificaram ausência de planos de saúde individuais, número insuficiente de enfermeiros escolares ou educadores treinados para atender às necessidades decorrentes do diabetes, além da inadequação dos serviços nutricionais ou informações nutricionais fornecidas aos familiares pelas escolas, de modo a permitir o planejamento das doses de insulina necessárias.

Essas crianças precisam que os educadores escolares tenham conhecimentos e competências básicas para garantir a segurança do ambiente escolar.^6^ Os pais e as equipes de saúde responsáveis por essas crianças devem trabalhar juntos, para munir o sistema de educação e outros cuidadores com as informações necessárias, de modo que as crianças possam participar totalmente e de maneira segura das atividades pedagógicas e de lazer proporcionadas pelas escolas.^7^

Para assegurar a segurança e apoiar essas crianças/jovens diabéticos, as escolas devem ter uma série de políticas, como: se existir um ou mais alunos diabéticos, as escolas devem assegurar que pelo menos dois de seus colaboradores sejam treinados para oferecer suporte; assegurar que exista uma área para autocuidado da criança diabética, incluindo monitoramento da glicemia, onde a privacidade dos alunos seja preservada; desenvolver um plano individual de saúde para cada aluno, discutido com os pais, o diretor e os educadores; e assegurar que todos os professores ou supervisores saibam reconhecer e tratar a hipoglicemia.^1 , 8 , 9^

## OBJETIVO

Avaliar o impacto do Programa Diabetes + Apoio dos Educadores Escolares às Crianças com Diabetes Tipo 1 (DARE+) sobre o nível de conhecimento e confiança dos educadores escolares para apoiar alunos diabéticos tipo 1; relacionar o nível de conhecimento dos educadores escolares com as variáveis acadêmicas e profissionais.

## MÉTODOS

Este estudo usou um desenho quasi-experimental de pré-teste/pós-teste, sem Grupo Controle.

A amostra foi composta por educadores escolares que trabalhavam com crianças diabéticas tipo 1. Do total de 12 escolas participantes, 129 educadores participaram no início do estudo (T0) e 113 no final do estudo (T1). Os dados foram coletados entre março de 2014 e março de 2015.

A intervenção envolveu as seguintes etapas:

- Considerando-se que o treinamento dos educadores escolares seria conduzido por enfermeiros de saúde escolar, a primeira etapa incluiu sua capacitação. O treinamento durou 21 horas, ao longo de 3 dias, com 14 horas dedicadas ao tópico de crianças/adolescentes com diabetes tipo 1.^9 , 10^
- Uma reunião preliminar foi realizada com o diretor das escolas, para apresentar os objetivos e obter autorização, para agendamento da intervenção.
- O Programa de intervenção DARE+ incluiu duas sessões, com um total de 6 horas e 1 semana de intervalo. Cada sessão incluiu uma parte teórica e uma prática, abordando os tópicos recomendados por associações nacionais e internacionais.^1 , 9 , 10^

Como parte dessa intervenção e para implementação do plano individual de saúde que contempla elementos indicados pela *American Diabetes Association* (ADA)^10^ e pela Direção Geral da Saúde (DGS),^9^ uma reunião foi realizada com os pais das crianças/jovens e os educadores responsáveis por prestar apoio a eles.

Os dados foram coletados por questionário constituído por três partes, autopreenchido:

- Habilitações acadêmicas e profissionais dos educadores escolares: profissão, qualificações acadêmicas, anos de experiência profissional, anos de experiência profissional na instituição, e tipo de vínculo empregatício.
- Questionário sobre conhecimento do diabetes,^11^ composto por 17 itens com três respostas possíveis (apenas uma é correta) e uma quarta opção “Não sei”.

Após receber autorização dos autores, o questionário foi validado em termos linguísticos e culturais, de acordo com as diretrizes internacionais.^12 - 14^

A consistência interna também foi determinada pelo valor de alfa de Cronbach, registrado como 0,926. Atribuiu-se 1 ponto a cada resposta correta e zero às respostas incorretas e respostas “Não sei”. A pontuação variou, assim, entre zero e 17 pontos.

- Questionário sobre a confiança dos educadores escolares para apoiar alunos com diabetes tipo 1.^11^ Esta escala contempla um escala Likert de 4 pontos, com cinco respostas possíveis (1 para discordo totalmente; 2 para discordo; 3 para não tenho opinião; 4 para concordo, e 5 para concordo totalmente). Os valores variam de 4 a 20 e, quanto maior o valor, maior o nível de confiança para apoiar alunos com diabetes tipo 1.

As características psicométricas foram determinadas, e o valor de alfa de Cronbach foi de 0,836.

A validade de construto foi testada pela análise fatorial exploratória com rotação Varimax e normalização de Kaiser. Apenas um fator foi obtido, o que explica a variância de 67,4%. Os valores da comunalidade flutuaram entre 0,670 e 0,714. O Kaiser-Meyer-Olkin foi de 0,799, e o valor do teste de esfericidade de Bartlett foi de 200,677 (p<0,001).

A análise estatística foi realizada com o programa (SPSS), versão 21.0. A frequência relativa e absoluta foi usada para as habilitações acadêmicas e profissionais dos educadores escolares.

Foram usados testes estatísticos não paramétricos de Correlação de Spearman (teste independente) e Wilcoxon (teste pareado), já que os dados não tinham distribuição normal (avaliada pelo teste de Kolmogorov-Smirnov). Como nem todos os grupos tiveram n≥30, o uso do teorema do limite central não foi utilizado.

Um nível de significância de 0,05 (p<0,05) foi adotado nas análises inferenciais.

Este projeto de pesquisa foi aprovado pelo Comitê de Ética em Pesquisa do Ministério da Educação (parecer no. 0396300001), tendo sido obtido o consentimento livre e informado de todos os participantes incluídos no estudo.

## RESULTADOS

Considerando que, em cada programa e cada escola, a intervenção envolveu pequenos grupos de educadores, foi decidido que não se perguntaria a idade e o sexo dos participantes. Houve duas razões para isso: essas informações não eram essenciais para os objetivos do estudo, e para impedir que os educadores fossem identificados, já que, em algumas escolas, apenas um homem participou do estudo.

Mais da metade (51,2%) dos participantes era professor, dos quais 13,6% tinham título de Mestrado/pós-graduação e já trabalhavam há mais de 10 anos na instituição, com vínculo empregatício ( Tabela 1 ).

**Tabela 1:** Habilitações acadêmicas e profissionais antes da intervenção (T0)

Variáveis acadêmicas e profissionais	n (%)
Profissão	
Assistente operacional	42 (32,6)
Professor	66 (51,2)
Outro (monitor, motorista, professor de educação infantil, cozinheiro)	21 (16,3)
Qualificações acadêmicas, anos	
6	15 (12,0)
9	11 (8,8)
12	20 (16,0)
Bacharelado/Ensino Superior	62 (49,6)
Mestrado/Pós-graduação	17 (13,6)
Experiência professional, anos	
<1	7 (5,6)
1-5	17 (13,6)
6-9	22 (17,6)
10-20	39 (31,2)
>20	40 (32,0)
Anos na instituição	
<1	13 (10,2)
1-5	30 (23,6)
6-9	25 (19,7)
10-20	34 (26,8)
>20	25 (19,7)
Detalhes do vínculo empregatício	
Contrato a tempo total certo	108 (83,8)
Contrato a tempo total incerto	2 (1,6)
Contrato a tempo parcial	10 (7,8)
Outros	9 (7,0)

Considerando que, quanto maior o valor, maior o nível de confiança para apoiar alunos com diabetes tipo 1, após a intervenção, em média (x̄=16,2 ±1,3; P_50_=16), os educadores escolares consideraram que seu nível de confiança para apoiar crianças diabéticas tipo 1 estava melhor do que antes (x̄=9,8±3; P_50_=9,8). Esta diferença foi significativa (Wilcoxon=-9,536; p<0,001). Apenas dez participantes não tiveram aumento em seu nível de confiança, sendo que, para oito deles, os valores permaneceram iguais, e dois tiveram valores menores.

Em relação ao nível de conhecimento, as diferenças entre T0 (x̄=10,8±2,8; P_50_=11) e T1 (x̄=13,7±2,1; P_50_=11) foram significativas (Wilcoxon=-7,914; <0,001), e 89 dos educadores escolares tiveram aumento em seu nível de conhecimento. Dos 113 educadores escolares que participaram da avaliação final, 89 (78,85%) tiveram aumento em seu nível de conhecimento.

Uma análise da relação entre o nível de conhecimento e o nível de confiança dos educadores escolares para apoiar alunos com diabetes tipo 1 antes da intervenção mostrou correlação baixa, positiva e significativa entre as duas variáveis (rs=220; p<0,05). Na medida em que o conhecimento aumentou, cresceu também o nível de confiança, para apoiar crianças/jovens diabéticos. Após a intervenção, a correlação continuou sendo fraca e negativa, mas não significativa (rs=-0,016; p>0,05).

Tanto antes (χ^2^=11,6; p<0,05) quanto depois da intervenção (χ^2^=13,1; p<0,05), o nível de conhecimento variou de acordo com as qualificações acadêmicas. Quanto maiores as qualificações acadêmicas, maior foi o nível de conhecimento dos educadores escolares sobre o diabetes tipo 1 ( Tabela 2 ).

**Tabela 2:** Aplicação do teste de Wilcoxon para nível de conhecimento sobre diabetes tipo 1 entre os educadores escolares, antes e depois da intervenção, de acordo com as respectivas qualificações acadêmicas

Qualificações acadêmicas	Antes da intervenção			Após a intervenção			Z	Valor de p
	x̄	Desvio padrão	Mediana	x̄	Desvio padrão	Mediana		
6 anos	9,7	2,5	9	11,9	3,3	13	-2,177	0,029
9 anos	8,0	4,2	10	13,0	1,8	13	-2,613	0,009
12 anos	10,9	2,1	10,5	13,6	1,8	14	-2,899	0,004
Bacharelado/Ensino Superior	11,4	2,3	12	14,2	1,7	15	-5,831	0,000
Pós-graduação/Mestrado	11,5	3,0	12	14,6	1,5	15	-2,662	0,008

A divisão da amostra de acordo com anos de experiência profissional mostrou que, antes da intervenção, o nível de conhecimento não se relacionava com o número de anos de experiência profissional (p>0,05). Após a intervenção, o nível de conhecimento (p<0,05) diferiu de acordo com o número de anos de experiência profissional.

Quanto mais anos de experiência, maior o nível de conhecimento, exceto nos grupos de profissionais com menos de 1 ano de experiência. Nesse grupo, não foi observado aumento no nível de conhecimento ( Tabela 3 ).

**Tabela 3:** Aplicação do teste de Wilcoxon ao nível de conhecimento sobre diabetes tipo 1 entre os educadores escolares, antes e depois da intervenção, de acordo com os anos de experiência profissional

Anos de experiência profissional	Antes da intervenção			Após a intervenção			χ^2^	Valor de p
	x̄	Desvio padrão	Mediana	x̄	Desvio padrão	Mediana		
<1	10,7	2	11	11,2	3,5	11	-0,816	0,414
1-5	11	2,7	12	12,8	1,9	13	-2,408	0,016
6-9	10,8	2,1	11	14	1,6	14	-3,899	0,000
10-20	10,7	2,6	11	13,9	1,9	14	-4,494	0,000
>20	11,3	2,9	11,5	14,4	1,6	14	-4,582	0,000

## DISCUSSÃO

Quando uma criança com diabetes tipo 1 é matriculada em uma escola, é importante que a instituição oriente e gerencie seu plano de ação em torno de dois pontos principais: treinamento dos educadores e desenvolvimento e aplicação do plano individual de saúde da criança.^8 - 10^ Este estudo também se concentrou nesses dois pontos cruciais.

A integração dos profissionais dos cuidados de saúde primários e hospitalar, e a cooperação entre eles também foram preocupações ao se definir a intervenção a ser implementada, conforme sugerido por diversos autores.^9 , 15^ Antes da intervenção, os enfermeiros de saúde escolar passaram por treinamento, envolvendo a equipe multidisciplinar de pediatria de um hospital, e a equipe multidisciplinar dos cuidados de saúde primários.

Os enfermeiros participaram na fase da intervenção juntamente dos educadores escolares. A intervenção durou 6 horas, ou seja, um período mais longo que as 4 horas recomendadas pela DGS.^9^

Todos os educadores escolares participaram da intervenção, incluindo professores, educadores de berçário, assistentes operacionais, motoristas, cozinheiros e recreadores culturais, que declararam que a educação em diabetes deve ter como público-alvo não apenas os professores, mas todos os profissionais que interagem com as crianças.^9 , 10^

Mais de metade da amostra (63,2%) tinha Ensino Superior completo. A mesma percentagem dos colaboradores tinha mais de 10 anos de experiência.

Em uma revisão da literatura,^2^ constatou-se que a maioria das crianças não recebia os cuidados necessários nas escolas. Wagner et al.,^16^ já tinham chegado a essa conclusão e, ao analisarem 132 educadores escolares, observaram que eles não estavam preparados para cuidar de crianças/jovens diabéticos.

Embora os estudos de Alnasir^17^ e Aycan et al.,^18^ tenham sido conduzidos apenas com professores, as conclusões confirmam o baixo nível de conhecimento desses profissionais para dar suporte a crianças diabéticas. Separando o nível de conhecimento em áreas, outros autores^6^ verificaram que o conhecimento dos sintomas era bom, mas o conhecimento das complicações e do tratamento do diabetes era insatisfatório. Alguns desses aspectos foram incluídos no Programa DARE+.

Para oferecer cuidados adequados, é necessário conhecimento, e o nível de conhecimento dos participantes deste estudo, antes da intervenção, foi de 63,7% da pontuação máxima possível. O valor é um pouco mais alto que o de outros estudos,^17 , 18^ embora estes tenham sido conduzidos apenas em uma amostra de professores. Após a intervenção do Programa DARE+, o nível de conhecimento dos educadores escolares melhorou, chegando a 80,6% da pontuação máxima possível.

As diretrizes da Direção Geral da Saúde afirmam que crianças muito novas precisam de mais cuidados, e escolas com educadores despreparados podem ficar em desvantagem. Portanto, os enfermeiros de saúde escolar precisam trabalhar em parceria com as crianças, pais e educadores escolares, para garantir que o tratamento/acompanhamento de crianças/jovens diabéticos esteja totalmente integrado ao cotidiano das escolas.^9^ Os estudos mostram que os educadores de escolas primárias preocupam-se com a administração de injeções de insulina e o monitoramento da glicemia e,^19^ assim, o treinamento adequado dos educadores escolares otimiza a segurança das crianças diabéticas.^19 - 22^

As conclusões indicam que, após a intervenção, os educadores escolares ficam mais confiantes do que antes para apoiarem crianças com diabetes tipo 1, e a diferença é estatisticamente significativa (p<0,01). Outros estudos^20 - 22^ também mostraram que houve aumento no conhecimento dos educadores escolares em relação a como cuidar de alunos com diabetes tipo 1, após a implementação de um programa educacional.

Por outro lado, Husband et al.,^11^ não constataram melhoria no conhecimento dos professores com o uso de uma metodologia de treinamento não interativo por CD-ROM. Eles recomendaram o uso de metodologias mais ativas nesse tipo de intervenção. Tais dados reforçam a decisão deste estudo de usar metodologias ativas, com a participação dos educadores escolares em atividades simuladas e discussões de casos.

Antes da intervenção, o nível de conhecimento não teve relação com o número de anos de experiência profissional (p>0,05). Wagner et al.,^16^ constataram também que os educadores escolares com 10 anos de experiência profissional e que tinham contato com crianças/jovens diabéticos não estavam preparados para cuidar deles. A participação no Programa DARE+ levou à melhora no nível de conhecimentos dos educadores escolares (p<0,05). Apenas o grupo de profissionais com menos de 1 ano de experiência não apresentou melhoria no nível de conhecimento.

Tanto antes (χ^2^=11,6; p<0,05) quanto depois (χ^2^=13,1; p<0,05) da intervenção, o nível de conhecimento variou de acordo com as qualificações acadêmicas. Quanto maiores as qualificações acadêmicas, maior o nível de conhecimento dos educadores escolares sobre diabetes tipo 1.

É importante salientar que, em todas as escolas, um plano individual de saúde foi definido/aprimorado para cada criança/jovem, permitindo que todos eles tivessem um educador para lhes prestar apoio relativo aos cuidados do diabetes durante o horário escolar. Embora considerado uma boa prática e recomendado,^1 , 8 - 10 , 23^ esse plano não representa a realidade, já que os dados de um estudo de Driscoll et al.,^22^ nos Estados Unidos, mostraram que 57% das crianças não tinham ninguém na escola encarregado de acompanhá-los em seus cuidados com o diabetes durante o horário escolar.

Para além da avaliação de conhecimentos e confiança dos educadores, há necessidade de avaliar a curto, médio e longo prazo os efeitos nas crianças, nomeadamente na qualidade de vida, qualidade de vida acadêmica, número de incidentes, rendimento acadêmico e absentismo na escola.

É também necessário garantir o treinamento/capacitação contínuo dos enfermeiros que treinam os educadores escolares e também dos próprios educadores, já que são eles que prestam cuidados diretos e imediatos às crianças em situações de emergência.

Nesse contexto e tendo em vista que a implementação de planos envolvendo os principais atores é essencial, embora não seja fácil realizar estudos randomizados, estes são importantes para validação, não apenas do programa de intervenção, mas também dos recursos envolvidos no programa.

Os resultados do estudo reforçam a necessidade do desenvolvimento de recomendações e medidas políticas, no sentido de os serviços de saúde, principalmente de saúde escolar, implementarem ações de capacitação dos educadores escolares, uma vez que se verifica que ações estruturadas com foco na capacitação dos educadores melhoram seu conhecimento e a perceção de melhoria quanto à resposta que podem oferecer ao estudante com diabetes tipo 1.

A definição de um programa estruturado, com sua avaliação e os resultados que agora se produzem, reforçando a eficácia da intervenção, pode influenciar/melhorar as práticas, uma vez que se define um modo de atuação *standard* , integrado, com envolvimento de todos os elementos-chave: família, estudante, equipe de cuidados da área de especialidade, equipe de saúde escolar e escola.

Este estudo contribui para as pesquisas neste campo, permitindo identificar estratégias integradas de apoio à criança e ao jovem com diabetes tipo 1. Novas conclusões levantam novas questões relativas ao cuidado em contexto escolar do aluno com diabetes tipo 1, dando continuidade a investigações anteriores e estimulando novas investigações para clarificar que estratégias funcionam melhor, com diferentes grupos, ou em diferentes circunstâncias.

As limitações do estudo incluem tamanho da amostra, modo de recrutamento e localização geográfica limitada da amostra. Ainda são necessários estudos experimentais, com Grupo Controle e com amostras maiores e randomizadas.

Outra limitação foi que não houve acompanhamento dos educadores escolares, e nem análise do impacto nos ganhos de qualidade de vida dos alunos e familiares, principalmente em relação aos principais indicadores de melhora no manejo da doença e, consequentemente, melhor acompanhamento de crianças/jovens diabéticos.

## CONCLUSÃO

A intervenção Diabetes + Apoio pelos Responsáveis Escolares às Crianças com Diabetes Tipo 1 (DARE+) conseguiu efetivamente melhorar o conhecimento e a autopercepção de quão preparados os educadores escolares estão para apoiar crianças/jovens com diabetes, sendo importante destacar algumas sugestões e implicações relativas à prática e à pesquisa.

Intervenções para melhorar os conhecimentos e confiança são importantes a curto prazo, sugerindo-se a realização de *follow-up* , assim como a formação e o apoio continuado aos educadores realizado pelos enfermeiros, permitindo que os conhecimentos sejam constantemente reciclados.
